# Efficacy effects of fecal microbiota transplantation on depressive symptoms: a meta-analysis based on randomized controlled trials

**DOI:** 10.3389/fpsyt.2025.1629290

**Published:** 2026-01-29

**Authors:** Jiamin Fu, Yuchi Zhang, Jing Gao, Ming Lan, Zhixin Zhang, Ruiting Liang, Hongyun Zhou, Shirui Liu, Ziyi Zhou, Jinwan Zhao, Xing Yu, Yuewen Liu, Peipei Han, Xiaoyu Chen, Cheng Lin, Qi Guo

**Affiliations:** 1 Department of Rehabilitation Medicine, Shanghai University of Medicine and Health Sciences Affiliated Zhoupu Hospital, Shanghai, China; 2 School of Sports and Health, Tianjin University of Sport, Tianjin, China; 3 Department of Rehabilitation Medicine, Shanghai University of Medicine and Health Sciences, Shanghai, China; 4 General Practice Clinic, Pujiang Community Health Service Center in Minhang District, Shanghai, China; 5 Department of Rehabilitation Medicine, School of Health, Fujian Medical University, Fujian, China; 6 Fujian Medical University, Fuzhou, Fujian, China

**Keywords:** depression, efficacy, fecal microbiota transplantation, gastrointestinal microbiota, intestinal microflora, meta-analysis

## Abstract

**Background:**

The gut microbiota plays a crucial role in the bidirectional communication between the gut and the brain. Although there has been much discussion in recent years on the link between depression and fecal microbiota transplantation (FMT), its effectiveness in treating depression remains debatable. The purpose of this study was to examine if FMT alleviates depression or its symptoms and to examine the possible impacts of various demographic subgroups and supplementation techniques.

**Methods:**

A systematic search of articles in the database (PubMed, EMBASE, Web of Science, and Cochrane libraries) before March 2025. The Revman 5.3 software was used to incorporate standardized mean difference (SMD) and assess quality of evidence using recommended grading assessment, development and evaluation tools to investigate whether FMT efficacy on depressive symptoms or depression.

**Results:**

This meta-analysis included seven studies involving 235 subjects, and the results did not support a significant treatment effect of FMT on depression [SMD: -0.10; 95%CI: (-0.60,0.41); p = 0.71]. Subgroup analysis showed the correlation between the intervention effect of FMT and confirmed depression at baseline, intervention dose, donor source, repeated route of drug administration, and dietary habits of subjects.

**Conclusions:**

There is insufficient evidence to support a significant efficacy of FMT on depressive symptoms, but subgroup analyses suggest that FMT may have a better antidepressant effect in the population with confirmed depression, and therefore more randomized controlled trials are necessary to validate the association between FMT and depressive symptoms.

## 1 Introduction

Depression is a serious mental illness that poses a significant public health challenge worldwide, not only due to its impact on individuals but also as a primary catalyst for suicide (1, 2). By 2030, depression could overtake heart failure to become the most prevalent disease in the world, according to research (3).Depression is not just a mood disorder, as a complex disorder characterized by a range of emotional, physical and cognitive symptoms, including but not limited to insomnia or lethargy, persistent fatigue, loss of appetite and mood swings, when severe as a potential threat to life (4, 5).In addition to the adverse effects on the individuals affected, these disorders place a significant financial strain on society due to high healthcare costs. These points make it evident that there is a need for effective treatments (6).

The human gastrointestinal tract inhabits trillions of microorganisms, including bacteria, viruses, archaea, and fungi, and is an important part of maintaining human health (7). There are significant variations in the individual bacterial content at birth because gut microbial colonization is a dynamic system that exhibits life stage-specific dynamics as well as being impacted by genetics, nutrition, metabolism, age, location, antibiotic therapy, psychiatric medicines, and stress (8–10). The “microbe-gut-brain axis” (MGB axis) refers to the significant role that the gut microbiota plays in the two-way communication between the gut and the brain. By controlling neuronal circuits and modifying the release of neurotransmitters inside the central nervous system, the gut microbiota affects an individual’s emotional equilibrium and offers fresh perspectives on the molecular underpinnings of mood disorders (11). The gut microbiota’s intricate complexity and dynamic nature exert significant influence on mental health, particularly in the context of depression. Among the interventions targeting the brain-gut axis, fecal microbiota transplantation (FMT) stands out as a transformative approach. FMT rapidly reconstitutes a patient’s gut microbiota by transplanting fecal microbiota from a healthy donor, typically administered via endoscopy, enema, or oral delivery of lyophilized material. While FMT is well-established for treating gastrointestinal disorders (e.g., chronic constipation, diarrhea, irritable bowel syndrome, inflammatory bowel disease, and functional bowel diseases), its therapeutic potential in neuropsychiatric conditions—including depression, autism spectrum disorders, anxiety, and Parkinson’s disease—is gaining increasing recognition (12, 13). FMT has shown good therapeutic potential to improve depression in preclinical studies (14, 15). In human clinical trials, FMT is a promising but initial intervention for the treatment of depression, and relevant intervention trials are relatively lacking, and its effect on human body is relatively vague (11). Currently, the treatment of depression primarily relies on traditional psychological therapies and pharmacological interventions. However, these approaches fail to adequately address the needs of many patients, with approximately 20–60% of individuals with depression exhibiting treatment resistance. Moreover, the side effects and adverse drug reactions associated with antidepressant medications often cause significant distress among patients (16, 17). Exploring more convenient and effective therapeutic strategies for depressive disorders remains a subject of active investigation. FMT as a gut microbiota modulation approach, holds promise as a potential alternative to traditional antidepressants, thereby offering novel insights into the biological mechanisms underlying mood disorders.

Although some articles have reported on the adverse effects and complications of FMT therapy (18),the application of FMT is gaining traction in both scientific research and clinical practice. Notably, the oral administration of lyophilized materials offers a less invasive and more standardized approach, achieving comparable therapeutic effects. However, as of 2020, only one systematic evaluation of FMT’s efficacy in psychiatric disorders has been conducted (7). This study focused on symptoms of mental illness, with outcome measures including various psychological test scores rather than specific depression scales, leading to significant heterogeneity in the results. This highlights the need for more robust and standardized outcome measures to fully assess FMT’s potential in treating depression and other psychiatric conditions In addition, the study was a narrative review with its own limitations, and it did not do a meta-analysis of the improvement of psychiatric symptom scale scores before and after the FMT intervention. Given the aforementioned considerations and the growing body of new randomized controlled evidence in recent years, we conducted a new systematic evaluation and meta-analysis for FMT intervention to improve depressive symptoms or depression related randomized controlled trials. The objective is to assess the efficacy of the depression scale score in comparison to a placebo.

## 2 Information and methods

### 2.1 Registration and reporting

The literature search, screening, inclusion and reporting were based on the systematic review and the Preferred Reporting Items for Meta-analysis (Preferred Reporting Items for Systematic Reviews and Meta-Analyses2020, PRISMA 2020) guidelines (19). Registration was registered in the International Prospective Systematic Review registry (PROSPERO) database (accession number CRD42024608863).

### 2.2 Inclusion and exclusion criteria

The inclusion criteria were as follows:

1. The included study was a randomized controlled trial;
2. Subjects with or without depressive symptoms or depression;
3. The control group did not take any other drugs that changed the intestinal environment (prebiotics, probiotics, antibiotics, etc.) at baseline or at the end of the intervention;
4. The participants were assessed at the depression assessment scale of the intervention based on the psychological symptoms of the participants.

The exclusion criteria are as follows:

1. Studies conducted in animal models;
2. Studies not published in English;
3. Studies comparing the effects of fecal microbiota transplantation on baseline and terminal outcome only in experimental groups with no control group;
4. Just a study protocol or a study that reports no results;
5. The intervention includes other nutritional supplements/drugs that alter the gut microbial environment;
6. Studies unable to obtain the full text or extract available data;
7. Repeated published studies (the latest published or the most complete study will be selected for inclusion).

### 2.3 Retrieval strategies

Two researchers (FJM and ZYC) independently conducted a systematic search of the PubMed, EMBASE, Web of Science and Cochrane libraries and covered all possible relevant articles to assess the improvement effect of FMT on depressive symptoms. The time range of literature search was from the database establishment until March 2025. Title and abstract based identification using available data in each database. The full text was evaluated only for studies defined as potentially eligible, following a title-and abstract-based procedure. To obtain the full text of the study, the corresponding author was requested for those not obtained in the above databases and libraries. Identification at all stages were performed independently by two researchers, but if a disagreement occurred, a third researcher (LAN Ming) was required to express their opinion. See Annex I for the complete search strategy of each database.

### 2.4 Data extraction

The retrieved articles were screened independently by two authors and data were extracted following a predesigned data extraction table. Any disagreement is resolved through a third-party discussion. Data extracted from each study included: author; year of publication; country; duration of follow-up; donor characteristics of fecal microbiota; characteristics of experimental and control groups (e. g., mean age, sex, sample size, presence, presence of other diseases or medications, changes in gut microbiota before and after FMT intervention); followed FMT intervention protocol; trial registration number; mean and standard deviation of participant depression assessment scale scores at baseline, terminal, and between. The scales used in different studies to assess psychological symptoms were not uniform, and when multiple depression assessment scales were used in the same study, the primary outcome measures or the more well-known and more commonly used scales in the original study were prioritized. Pre-post score changes were assessed by summarizing the standardized mean difference (SMD) to find associations between the FMT intervention and improvement in depressive symptoms.

### 2.5 Quality assessment and outcome measurement

We assessed the quality of individual trials using the Cochrane Collaborative Risk of Bias tool, which summarizes the risk of bias across items (20). These included information on random sequence generation (selection bias), allocation concealment (selection bias), subject and researcher blindness (performance bias), outcome assessors blindness (detection bias), processing incomplete data (loss-to-visit bias), and selective reporting of initially mentioned results (reporting bias). After examining the full text of the included articles, the authors classified the experimental risk into three levels: high risk, unclear risk, and low risk based on the above parameters. Two researchers (ZYC and LM) independently assessed the risk of bias, and any differences were resolved through consultation with Fu Jiamin.

The efficacy outcome measures for this meta-analysis were the differences from baseline to terminal depression scores between the experimental and control groups, and these differences were assessed by multiple depression assessment scales.

### 2.6 Statistical analysis

Each study of the total difference in changes in depressive symptoms between the FMT and control groups was combined by using the Revman 5.3 software to assess the efficacy of FMT on depressive symptoms. With the data extracted from each study, the effect size was calculated as a standardized mean difference with a 95% confidence interval (CI) for the continuous variables.

For the whole sample, heterogeneity tests were estimated using the Cochran chi-square test, and discordance tests were used. Considering the differences in FMT intervention protocol and depression assessment scales, we applied a random effect model. All probabilities (p-values) of the data were two-sided and p <0.05 was considered statistically significant.

Potential variables leading to sources of heterogeneity were studied by subgroup analysis. Potential variables included: FMT intervention dose, donor source, repeat administration, and route of administration; whether subjects were depressed at baseline, eating habits, and different depression scales used in the study. Publication bias will be assessed using funnel plots when included in study 10.When fewer than 10 studies were included, the Egger’s test was performed using Stata 17 software to assess publication bias. In addition, the software was used for sensitivity analysis to estimate whether it affects the pooled effect by excluding each study in turn.

## 3 Result

### 3.1 Study selection

**Figure 1:**
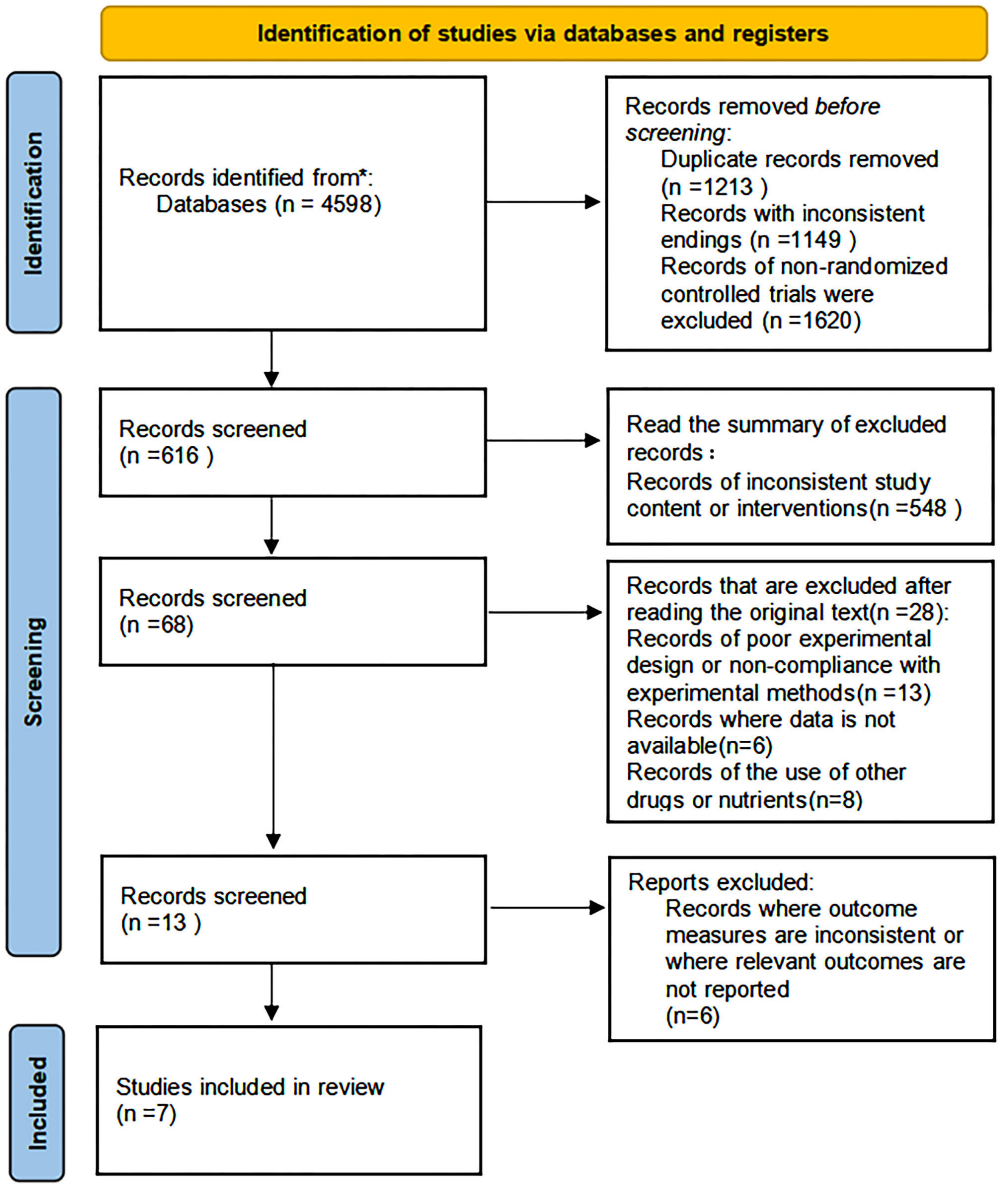
Flow chart of the literature.

A total of 4598 related studies were evaluated after the database search. After removing 1213 duplicates and 1620 non-RCT studies, 576 studies were excluded by screening the titles and abstracts. Of these, 548 study programs were excluded due to inconsistent study content or intervention/control measures, 13 studies were excluded due to poor experimental design or inconsistent experimental methods, 6 studies were excluded because data were not available, and 8 studies were excluded for supplementation of other drugs or nutrients. Furthermore, in the full-text evaluation and evaluation of the remaining 13 studies, six study programs were excluded due to the inconsistency of outcome measures with the present study and not reporting relevant results. Ultimately, seven eligible studies were included based on the inclusion and exclusion criteria (**Figure 1**).

### 3.2 Study characteristics

**Table 1** summarizes the baseline data from the seven randomized controlled trials conducted in different countries and regions, including China (two studies) (21, 22), one study each in European countries (Finland and Norway) (23, 24), Canada (25), Australia (26) and the United States (27) **Table 1**. Study sample sizes were all small with a minimum of 15 and a maximum of 54. The follow-up time varied greatly among the studies, with the shortest being only 8 weeks and the longest reaching 52 weeks. In these studies, the scales used to assess depression symptoms included the Montgomery-Asperger Depression Scale (MADRS) (25, 26), the Hamilton Depression Scale (HAMD) (22), the Patient Health Questionnaire (PHQ-9) (21), the Hospital Anxiety and Depression Scale (HADS) (24, 27) and the Beck Depression Scale (BDI) (23). Of these, two studies (21, 25) used multiple scales to assess depressive symptoms simultaneously, and we extracted scores for the primary outcome measures. For the mean and SD values of the post-intervention scores for depressive symptoms, the two studies were obtained from chart estimates (22, 23). In seven studies, three of the studies were IBS patients who met the Rome III diagnostic criteria (22, 24, 27), the subjects included in both studies had a confirmed depression diagnosis at baseline (22, 26), another study included subjects with patients with Parkinson’s disease (21); The findings indicate that three studies support a significant improvement in depressive symptoms with the FMT (22, 24, 25),one study suggest that FMT works only in the short term, but no improvement in the long term (23),the results of the remaining three studies showed that FMT had no significant effect on depressive symptoms (21, 26, 27).

**Table 1:** Characteristics of the studies included in this meta-analysis.

Author, year	Country	Sample size (experimental group / control group)	Donor baseline characteristics	Receptor baseline characteristics	Follow weeks	Depression scale	Main findings and conclusions	Secondary outcome	Treatment of depression efficacy
Yasaman GhorbaniBSc etl 2022 (25)	Canada	28(15/13)	18–45 years old, BMI 18.5–23 kg/m ^2^, HOMA-IR <2.73	average age 45.0 years(33.0-57.0), 87% female; BMI 35 kg/m², HOMA-IR of> 2.73	12	HAMA, MADRS	significant reduction in depressive symptoms	allogeneic FMT group showed favourable changes in gut microbiome composition, metabolites, pathway representation, and network analysis	positive
Faisabilité etl 2023 (26)	Australia	15(10/5)	NA	average age 44.09 ± 6.83 years, 60% female; MADRS score ≥20	8	MADRS, DASS	no significant difference between the FMT group and the placebo groups	The mean GI symptom score improved from the baseline to week 8, and the quality of life improved in the FMT group	negative
Yi Cheng etl 2023 (21)	China	54(27/27)	NA	average age 61.57 years, 40.74% female; mild to moderate PD	12	PHQ-9, GDS-15	no significant improvement in depressive symptoms	compared with the control group, FMT group improves MDS-UPDRS, IBS-RS, GSRS, and IBS-QOL scores, and cognitive function	negative
Olga C Aroniadis etl 2019 (27)	America	45(22/23)	NA	average age 37.5 years, 37% female; IBS-D patients	12	IBS-QOL, HADS	12 weeks of treatment did not yield significant efficacy and it was decided to terminate the trial	compared to the placebo group, IBS-QOL, HADS, and BSFS scores at 12 weeks no improvement	negative
Hao Lin etl 2021 (22)	China	18(9/9)	A 36-year-old healthy male with no smoking and drinking habits had a normal BMI	control group average age 50.44 years and FMT group 44.33 years; IBS-D patients HAMA score 14-24,HAMD score 20-34	12	HAMA和HAMD, IBS-QOL	alleviate the anxiety and depressive behaviors in patients with IBS-D	FMT treatment can improve the patients' quality of life	positive
Perttu Lahtinen etl 2019 (23)	Finland	49(23/26)	Adult male, good health, normal weight, no antibiotics in the past year, not healthcare worker	average age 47 years; 52.1% female in the FMT group, 65.3% female placebo group	52	BDI	Transient symptom relief	Changes tered microbiome in the FMT group and decreased fecal water content	Short-term positive, long-term negative
Tarek Mazzawi etl 2022 (24)	Norway	26(11/15)	Healthy family members living in the patient's family, male and female, over 18 years	average age 36 years, 74.4% female; IBS-D patients	24	HADS	Improvements in depressive symptoms	There was no significant difference in total FMTHAD scores, HADS, and HAMD scores after transplantation	positive

BMI, Body Mass Index; HOMA-IR, Homeostatic Model Assessment of Insulin Resistance; HAMA, Hamilton Anxiety Rating Scale; MADRSMontgomery-Asberg Depression Rating Scale; DASS, The Depression Anxiety Stress Scale; MDD, Major depressive disorder; PHQ-9, Patient Health Questionnaire-9;GDS-15, Geriatric Depression Scale-15; MDS-UPDRS, MDS-Unified Parkinson's Disease Rating Scale; IBS-SS, Irritable Bowel Syndrome Symptom Severity Scale; IBS-QOL, Irritable Bowel Syndrome Quality of Life; HADS, Hospital Anxiety and Depression Scale; HAMD, Hamilton Rating Scale for Depression; BDI, Beck Depression Inventory; IBS-D, Irritable Bowel Syndrome with Diarrhea; NA, Not Applicable.

All of the seven studies included in this paper used FMT as an intervention to improve depressive symptoms. **Table 2** details the specific intervention methods of FMT in each study subject, including administration route, dose, administration frequency, FMT preparation method, and placebo type **Table 2**. These studies were administered in a variety of ways, including oral capsule, gastroscopy, colonoscopy and enema. The dose of FMT ranged from 30 g to 50 g and was not explicitly mentioned in some studies (22, 25). In terms of administration frequency, three studies used a single dose (23–25),while the remaining four studies used repeated administration (21, 22, 26, 27). The preparation methods of FMT included single donor and multiple donor, including 5 studies selected single donor preparation (22–26)and 2 studies selected multiple donor (21, 27). In addition, 5 studies used frozen feces (21–23, 26, 27), and two studies used fresh feces (24, 25). The placebo type are abundant, including autologous FMT, blank capsule, pigment mixing, and saline.

**Table 2:** Characteristics of the FMT intervention mode in each study.

Author, year	Route of medication	Dose, form (stool / suspension)	Frequency of administration	FMT preparation (single / multiple supply) (fresh / frozen)	Placebo (autologous / non-FMT) (fresh frozen)
Yasaman GhorbaniBSc etl 2022 (25)	colonoscope	NA	single-dose	Single-donor / fresh feces	Self-derived FMT / fresh stool
Faisabilité etl 2023 (26)	enema	4 enema administration; total 50 g of stool	drug administered over 4 consecutive days	Single-donor / frozen stool	Non-FMT / (50 mL total saline, 10% glycerol and brown dye)
Yi Cheng etl 2023 (21)	capsule	50 grams of feces	Repeat 3 times (once per week)	Multiple-donor / frozen stool	Non-FMT
Olga C Aroniadis etl 2019 (27)	capsule	25 capsules per day for 28 g	Drug administration was repeated within 3 days	Multiple donor (frozen stool)	Non-FMT
Hao Lin etl 2021 (22)	capsule	30 per time	Repeat 3 times (once every 2 days)	Single-donor / frozen stool	Blank capsule
Perttu Lahtinen etl (23) 2019	colonoscope	30 g of feces; a suspension	single-dose	Single-donor / frozen stool	Autogenous FMT / frozen stool
Tarek Mazzawi etl 2022 (24)	gastroscope	30g of stool; suspension / 60ml	single-dose	Single-donor / fresh feces	Self-derived FMT / fresh stool

The effect of FMT on gut microbiota in depression is summarized in **Table 3**. In the seven included studies, FMT had a significant effect on the composition and abundance of the gut flora, but these changes showed some variation between the several included studies. The abundance of certain flora (such as Coprococcus, Bifidobacterium, Bacteroides, Roseburia, Eubacterium, etc.) increased significantly in the gut after the intervention, indicating that FMT may help to improve the overall balance of intestinal flora. However, the relationship between these flora changes and improvement in depressive symptoms is not clear. Although some studies observed significant improvement in depressive symptoms after FMT intervention (22, 24), other studies did not find similar significant effects (21, 23, 25–27), the efficacy of FMT on depressive symptoms may be influenced by multiple factors (individual differences, baseline flora status, dose and administration of FMT).

**Table 3:** FMT intervention in depression.

Author, year	Flora	Transformation
Yasaman GhorbaniBSc etl 2022 (25)	At 1 month, the number of Coprococcu, Bifidobacterium, Bacteroides and Roseburia increased significantly in the intestine, while Streptococcus decreased. At 3 months, the number of Bacteroides and Blautia increased in medium	Although the change decreased at 3 months, the overall allogroup developed in a beneficial direction
Faisabilité etl 2023 (26)	NA	NA
Yi Cheng etl 2023 (21)	Eubacterium eligens, Eubacterium ventriosum, Clostridiales bacterium 42_27, uncultured Blautia sp, Clostridioides difficile, uncultured Clostridium sp, Roseburia hominis	Increased abundance in the FMT group
Olga C Aroniadis etl 2019 (27)	Bacteroidetes, Firmicutes, Prevotella	The FMT group had a higher abundance as compared to the baseline
Hao Lin etl 2021 (22)	Faecalibacteriu, Eubacterium, Escherichia, Bifidobacterium	Increased in the FMT group
Perttu Lahtinen etl 2019 (23)	16 The V3 – V4 region of the S rRNA gene	Increased in the FMT group
Tarek Mazzawi etl 2022 (24)	Actinobacteria spp.ifidobacteria spp.Alistipes onderdonkii	Increased in the FMT group

### 3.3 Assess the quality of evidence of each study

**Figure 2:**
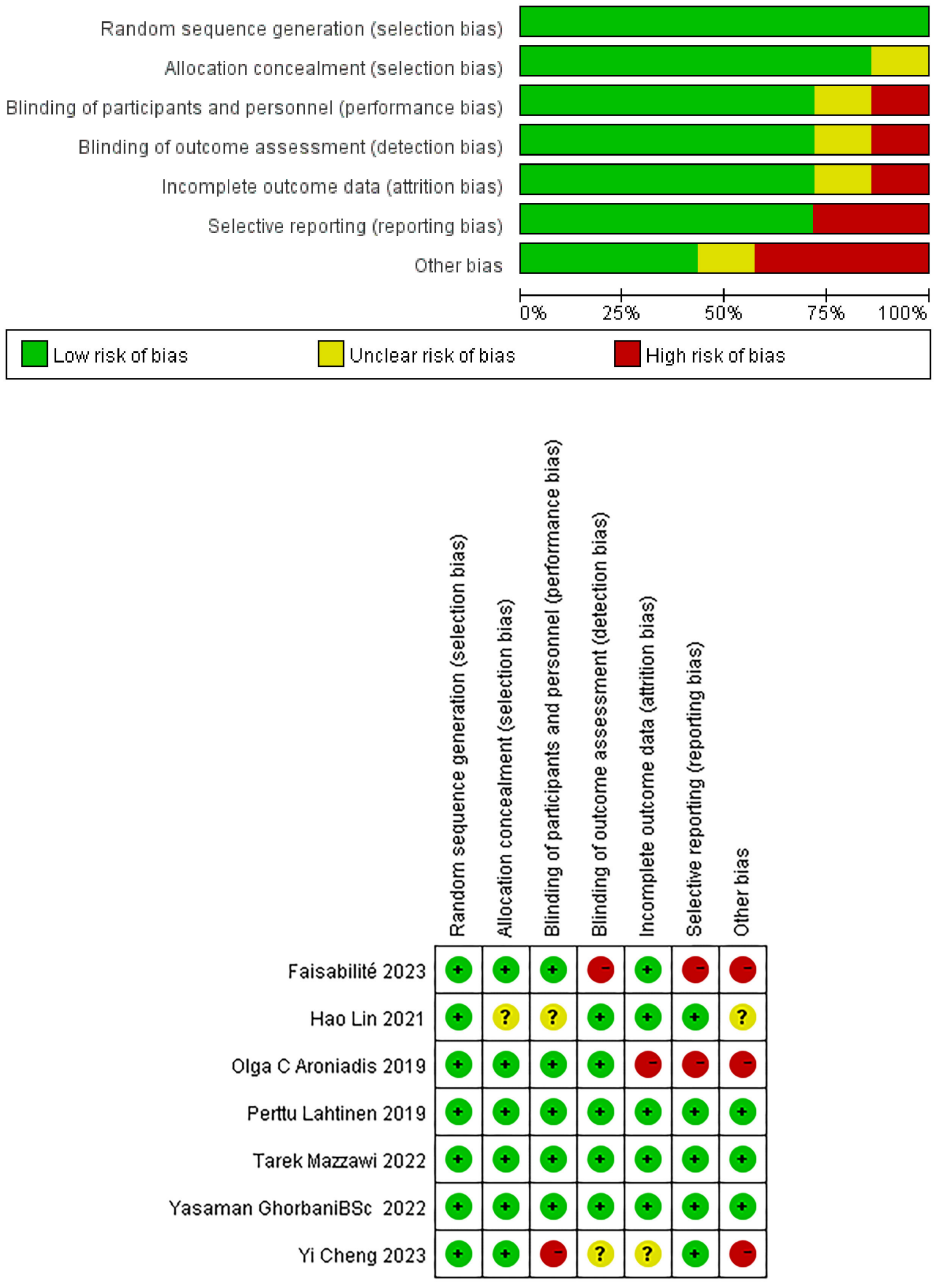
Quality assessment of the final screening study.

**Figure 2** presents the results of the quality assessment of the seven randomized controlled trials included in this study using the Cochrane Collaborative Risk of Bias Assessment tool. Three studies had high evidence quality (24, 25, 27) and no risk of bias, and four studies had obvious risk of bias (21, 22, 26, 27).Finally, according to the cochrane risk assessment criteria, three trials were assessed as high bias (21, 26, 27), and one trial was assessed as moderate bias (22).

### 3.4 Results of the meta-analysis

#### 3.4.1 FMT overall effect on the treatment of depressive symptoms

**Figure 3:**
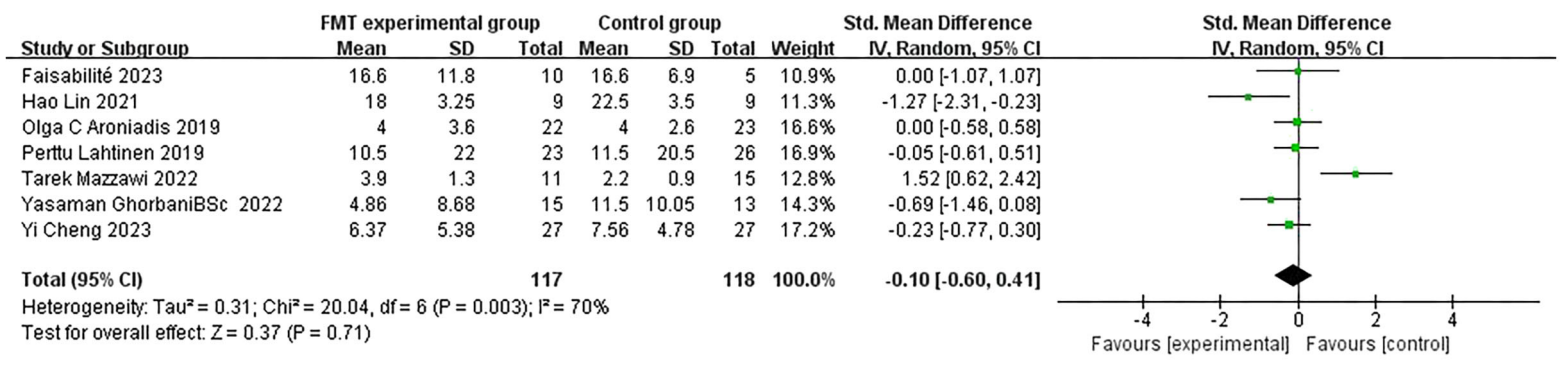
Forest plot of the association between fecal microbiota transplantation and Depression Scale scores compared to placebo.

Given the non-uniformity of depression rating scales used across the included studies, we employed a random-effects model for our analysis. A pooled analysis of seven studies revealed that FMT did not significantly affect depression scale scores [SMD: -0.10; 95% CI: (-0.60, 0.41); p = 0.71]. However, scores decreased from baseline to follow-up in the FMT group compared to the control group, suggesting that FMT may have an overall therapeutic impact on depression and could potentially serve as a means of alleviating depressive symptoms. Additionally, the results indicated high heterogeneity among the studies (I² = 70%; χ² = 20.04; p = 0.003), which may be attributed to differences in study design, participant characteristics, and outcome measures (**Figure 3**).

#### 3.4.2 Subgroup analysis

**Figure 4:**
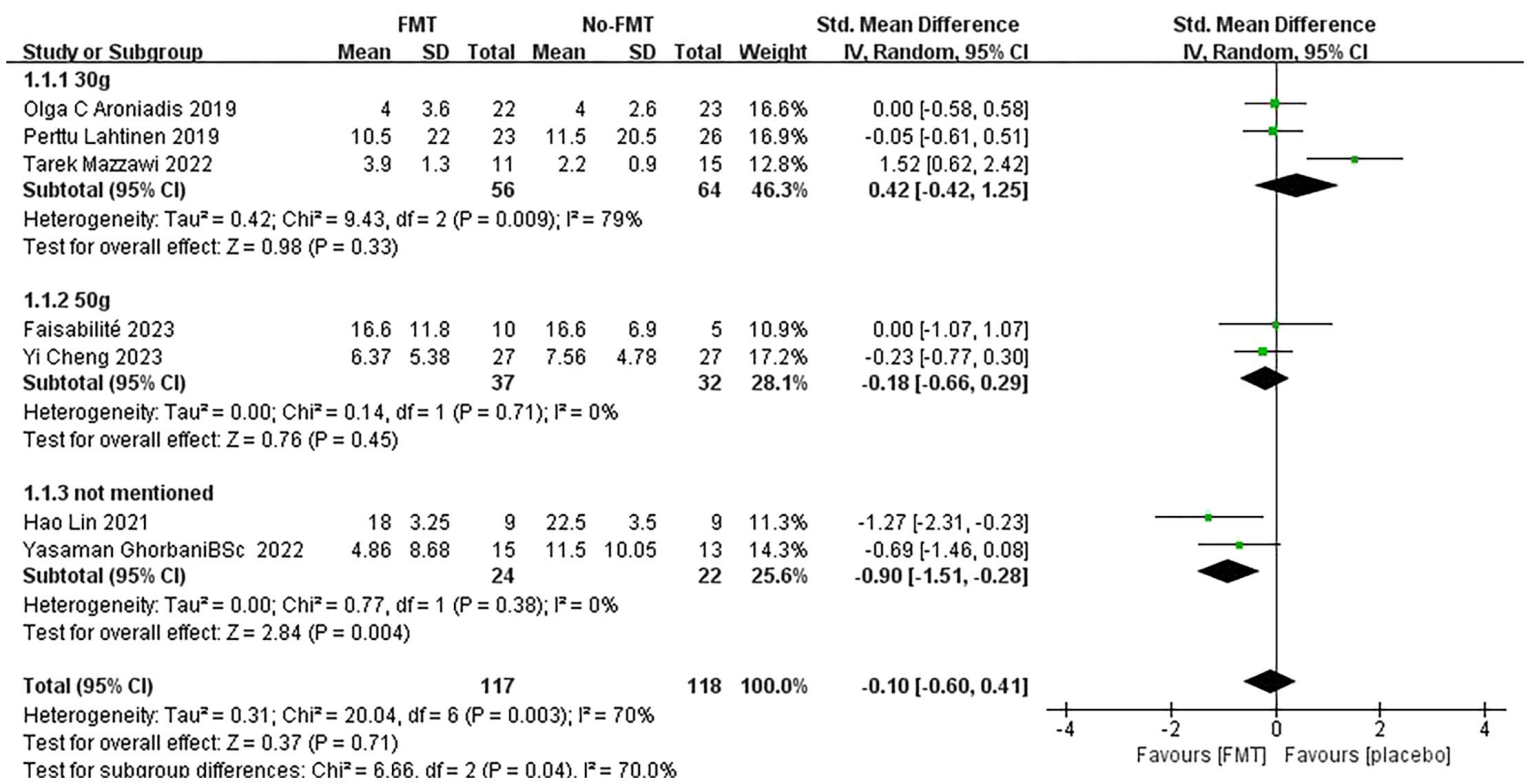
Forest plot of correlation between FMT and depression scale scores in different intervention dose populations.

**Figure 5:**
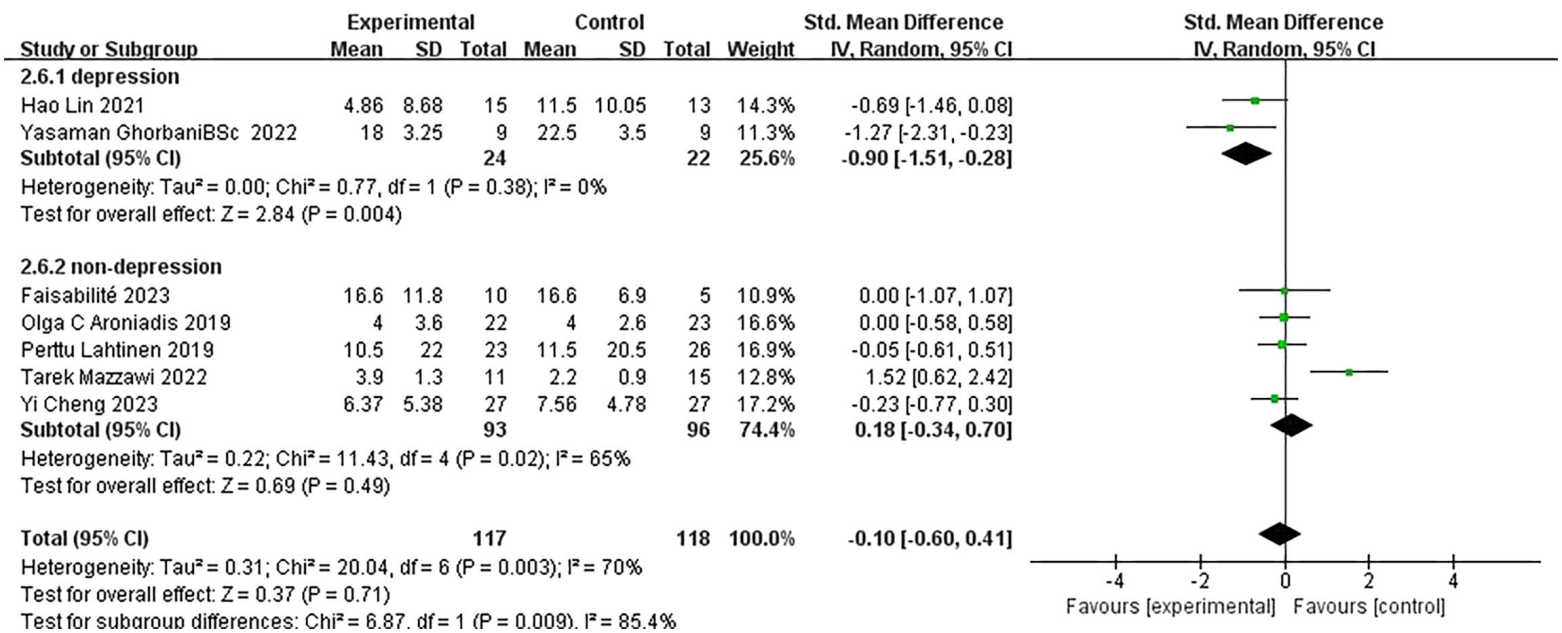
Forest plot of correlation between FMT intervention and depression scale scores in people with different baseline mental health levels.

In the subgroup analysis, three studies involving 120 subjects who received a 30g FMT intervention showed no significant difference between FMT and placebo [SMD: 0.42; 95% CI: (-0.42, 1.25); p = 0.33]. In contrast, four studies with higher FMT doses suggested better antidepressant efficacy, though the results were not statistically significant [SMD: -0.18; 95% CI: (-0.66, 0.29); p = 0.45]. Notably, two studies did not report the FMT intervention dose but showed a significant antidepressant effect compared to the placebo group [SMD: -0.90; 95% CI: (-1.51, -0.28); p = 0.004]. Interestingly, both of these studies included subjects diagnosed with depression, whereas the other five studies involved subjects without depression at baseline, in which FMT did not demonstrate a significant antidepressant effect [SMD: 0.18; 95% CI: (-0.34, 0.70); p = 0.49]. These findings suggest that FMT may have a more pronounced impact on individuals with a confirmed depression diagnosis, while its efficacy in non-depressed populations remains unclear. The observed heterogeneity (I² = 70%; χ² = 20.04; p = 0.003) highlights the need for further research to explore the factors contributing to these variations in outcomes (**Figure 4**, **Figure 5**).

Different FMT protocols may influence depression scores. For instance, repeated FMT administrations showed a trend toward greater improvement in depression scores compared to single-dose interventions [SMD: -0.28; 95% CI: (-0.74, 0.18); p = 0.23], although this difference was not statistically significant. Regarding the route of administration, upper gastrointestinal delivery (e.g., oral or gastroscopy) demonstrated a modest antidepressant effect [SMD: -0.23; 95% CI: (-0.65, 0.19); p = 0.53], though statistical significance was not achieved. Dietary factors also appeared to play a role; in two studies conducted in rice-consuming regions, FMT was associated with greater reductions in depression scores compared to studies in wheat-dominant areas [SMD: -0.65; 95% CI: (-1.65, 0.35); p = 0.20], although this finding remained non-significant.

In terms of donor source, neither single-donor [SMD: -0.09; 95% CI: (-0.92, 0.74); p = 0.83)]nor multi-donor [SMD: -0.13; 95% CI: (-0.52, 0.27); p = 0.53)]FMT interventions demonstrated a significant effect on depression outcomes compared to placebo. The seven included studies used five distinct depression assessment scales, contributing to heterogeneity in outcomes. Notably, while studies employing the Hospital Anxiety and Depression Scale (HADS) showed no significant antidepressant effect, those using alternative scales (e.g., the Beck Depression Inventory, Montgomery-Åsberg Depression Rating Scale) reported modest depressive symptom relief following FMT (**Table 4**).

**Table 4:** Subgroup analysis of the association of depression scores by FMT.

Subgroup Classification	Sample size	SMD	I2 (%)	P-value
FMT Intervention dose				
30g	3 (120)	0.42 [-0.42,1.25]	79	0.33
50g	2 (69)	-0.18 [-0.66,0.29]	0	0.45
NA	2 (46)	-0.90 [-1.51,-0.28]	0	**0.004**
Donor source				
Single donor	5 (136)	-0.09 [-0.92,0.74]	80	0.83
Multi-donor	2 (99)	-0.13 [-0.52,0.27]	0	0.53
Frequency of administration				
Single transplant	3 (103)	0.23 [-0.89,1.35]	86	0.69
Repeated administration	4 (132)	-0.28 [-0.74,0.18]	35	0.23
Route of medication				
Upper gastrointestinal tract (oral administration, gastroscope)	3 (92)	-0.23 [-0.65,0.19]	0	0.28
Lower digestive tract (enema, colonoscopy)	4 (143)	0.02 [-0.86,0.89]	83	0.97
Control group component				
Autologous FMT	3 (103)	0.23 [-0.89,1.35]	86	0.69
Non- FMT	4 (132)	-0.28 [-0.74,0.18]	35	0.23
**IBS at Baseline**	5 (166)	-0.08 [-0.81, 0.65]	80	0.82
**Depression at baseline**	2 (46)	-0.90 [-1.51, -0.28]	0	**0.004**
Food habits of staple food				
Rice	2 (72)	-0.65 [-1.65, 0.35]	67	0.20
Wheat	5 (163)	0.12 [-0.50, 0.74]	71	0.67
Depression scale				
MADRS	2 (43)	-4.30 [-9.93, 1.34]	18	0.20
PHQ-9	1 (54)	-1.19 [-3.90, 1.52]	N	0.39
HADS	2 (71)	1.05 [-0.57, 2.67]	62	0.20
HAMD	1 (18)	-4.50 [-7.62, -1.38]	N	**0.005**
BDI	1 (25)	-1.00 [g-12.96,10.96]	N	0.87
Sensitivity analysis				
Excluded high-offset risk studies	4 (121)	-0.11 [-1.12, 0.90]	85	0.83
Excluded shorter duration (<2 months)	6 (166)	-0.08 [-0.81, 0.65]	80	0.82
**Fixed effect model**	7 (235)	-0.10 [-0.36, 0.17]	70	0.46

Bolded value: p<0.05.

### 3.5 Publication bias and sensitivity analysis

**Figure 6:**
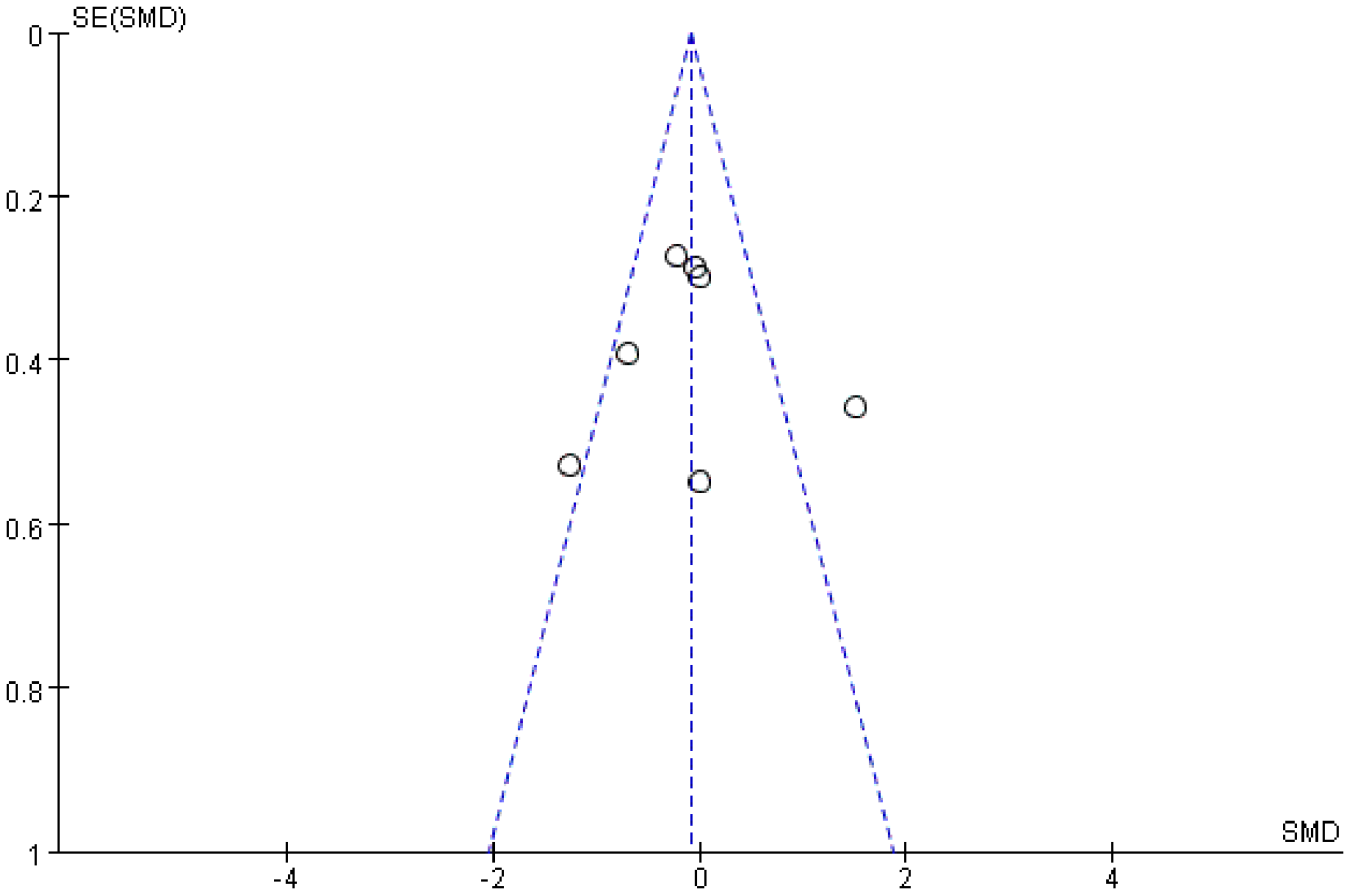
Funnel plot of FMT influence on depression scores.

To assess publication bias, funnel plots were generated for FMT’s improvement of depressive symptoms. The funnel plots in our results appear symmetrical around the effect estimates. Since fewer than 10 studies were included, we also performed an Egger test, yielding p=0.986 > 0.05, indicating no significant publication bias in our studies.(**Figure 6**).

The results of sensitivity analyses, by excluding studies with high risk of bias and short intervention duration and applying a fixed-effects model, support the stability of our results. In addition, we tested the sensitivity of our findings to each of the included studies by excluding studies one by one and found no impact on the stability of our results.

## 4 Discussion

This systematic review included previous randomized controlled trials investigating the efficacy of FMT on depressive symptoms, culminating in a summary analysis of seven trials. The results of the meta-analysis showed no significant association between FMT intervention and improvement in depression [SMD: -1.34; 95% CI: (-3.45, 0.77); p = 0.21]. Due to the high heterogeneity between studies, we stratified the studies according to the FMT intervention dose, source of donor, whether it was given repeatedly and the route of administration; whether the subjects were depressed at baseline, their dietary habits and the different depression scales used in the studies. Subgroup analyses showed that there was a more significant antidepressant effect using higher doses of FMT and that the depression efficacy of FMT was significant in those with a baseline diagnosis of depression. In addition, the intervention effect of FMT was correlated with the source of the donor, whether or not the administration route was repeated, the setting of the control group and the dietary habits of the subjects, but the results were still not significant.

Our findings run against the results of a previous systematic evaluation of the effects of FMT on the symptoms of psychiatric disorders. The systematic evaluation of Chinna et al (7)mainly focuses on various neuropsychiatric disorders possibly affected by the gut-brain axis, including depression, anxiety disorders, and autism spectrum disorder. Not only limited to the analysis and evaluation of depression patients and depression scale. Second, several studies have assessed the psychiatric symptoms or quality of life of IBS patients after undergoing FMT, and the Irritable Bowel Syndrome Quality of Life Evaluation Scale (IBS-QoL), the Irritable Bowel Syndrome Symptom Severity Questionnaire (IBS-SSS), and other disease prognostic assessment scales specific to patients with IBS used were not considered as a targeted in the screening process of the studies for our meta-analysis. So there was a crossover between this study and previous studies, and the included studies were not consistent. As a result, there is overlap between this study and earlier research, and the included studies are inconsistent. Lastly, the traits of the study participants, intervention techniques, and outcome analysis that each participating institute recruited are also strongly linked to the variations in the research findings. Furthermore, it should be noted that the study examining the psychological effects of FMT in IBS patients has certain limitations. This is because, although there is a clear improvement in mental symptoms, it is not possible to say that FMT will alleviate symptoms in patients with mental illness because the improvement in mental symptoms may be secondary to the improvement in gastrointestinal symptoms associated with IBS. FMT and emotional psychology could not be directly related.

When we explored sources of heterogeneity through subgroup analysis and meta-regression, we did not find that any subgroup significantly affected the stability of the results. However, we must consider the potential difficulties associated with using various assessment instruments. We had trouble guaranteeing the stability and consistency of the experimental results because of the disparities in objectivity and accuracy of these scales, which made us aware of the impact of different measurement instruments. Second, the effects of fecal transplant microorganisms on the recipient’s gut microbial ecology can also be influenced by the genetic backgrounds of both FMT donors and recipients, interactions between the gut microbiome, baseline health status, comorbidities, and drug use. For example, in people with depression and anxiety disorders, the gut microbes may be characterized by a high relative abundance of pro-inflammatory species, while the abundance of bacteria producing SCFA is low (28). Autoimmune diseases (such as psoriasis, purulent dermatitis, etc.) can cause gut microbial dysbiosis (29); Antibiotic use can limit the microbiota’s composition and drastically lower the number of bacteria in the feces. A particular antibiotic regimen can also cause depression by altering the gut microbiota. Although our subgroup analysis indicates that the result is robust and insensitive, it is necessary to take into account a number of more complex potential sources of variation.

It is yet unknown if microbial dysbiosis is a direct cause of depression or merely a result of its pathological alterations. Based on the analysis of earlier trials, we must take into account the potential influence mechanism of FMT on depression, even though the current data do not support the major effect of depression. The vagal nerve, neuroendocrine system, neuroimmune system, and autonomic nervous system are among the mechanisms that provide bidirectional communication between the gut bacteria and the brain. Numerous studies have demonstrated that disruption of the gut microbiota causes neurochemical alterations, including increased striatal monoamine turnover, decreased expression of serotonin receptors, decreased expression of synaptic plasticity genes, and changed levels of brain-derived trophic factor (BDNF) (30–32). This implies that in order to effectively avoid the onset and progression of depression and alleviate its symptoms, we need investigate suitable means of reestablishing the disturbed gut flora. On the other hand, depression may be triggered by low-grade inflammation brought on by abnormalities in the gut flora. The primary cause of inflammation that results in neurodegeneration may be the gut’s dysbiosis (33, 34). The gut microbiota can directly regulate the immune system, but in the case of bacterial dysbiosis, the cytokines of the related microorganisms can increase the intestinal permeability (35)and damage the blood-brain barrier (BBB). They can also activate microglia in the central nervous system (CNS) (36) and secrete pro-inflammatory cytokines that alter the structure and function of the brain (37), further leading to clinical depression. However, there are some key aspects of FMT intervention in depression that are not fully explored. First, the precise functions of microbial metabolites in the gut-brain axis and their impact on mood and brain function are not entirely understood, indicating the need for more focused research on which metabolites are involved and how they affect mood and brain function. The second area that requires more research is the role of the gut barrier in depression, how FMT affects its recovery, and how it relates to depressed symptoms. Lastly, it is important to consider the individual variability of intestinal flora and bacteria, as these may influence the specific microbial communities linked to depression and the disparities between hereditary and environmental variables (38). These research areas have not yet been fully explored. On the other hand, people susceptible to depression or depression also have a higher risk of disordered gut microflora than the general population. A prospective cohort study showed that the gut microbiota can be used as a predictor of antidepressant treatment outcomes for depression in older age (39). FMT may only have significant efficacy in people with bowel disorders caused by certain diseases, which may also be the reason why we found the antidepressant efficacy of FMT to be significant in our subgroup analyses of people with confirmed depression and did not find any significant effect of FMT in reducing depression scores in the nondiagnosed population. After decades of observational research, the field of the gut microbiome is shifting from association to regulation (40).The study’s ultimate objective is to encourage the creation of depression treatments based on microbiome. At this point, we should pay attention to both the potential influence of intestinal microflora on the effectiveness of antidepressants as well as the development of microbiota-targeted therapies.

The subgroup analysis of this study revealed that higher doses of FMT treatment were associated with better antidepressant efficacy, and the effect of FMT was significant in patients diagnosed with depression. However, the differences in the effect of FMT on depression treatment currently face the challenge of a lack of standardized treatment and evaluation protocols. Although most current studies have focused on analyzing changes in fecal microbial composition before and after FMT, there is a clear lack of research on the detailed description of pathogens and beneficial bacteria associated with depression and the mechanisms underlying their interaction. Additionally, the safety and potential ethical issues of FMT technology during the transition from clinical trials to clinical application cannot be ignored (41). Safety assessments need to consider genetic differences between donor and recipient and the potential biological risks associated with fecal transplantation, which requires the assessment of patient dietary habits, genetic characteristics, and compatibility of the microbial composition of the donor and recipient (42). Fine classification and metabolic analysis will provide better information to support clinical decision-making. Furthermore, the FMT procedure involves human samples and requires following compliant medical extraction procedures, including informed consent, privacy rights, and strict ethical and medical ethical standards (43–45). The application of FMT technology in the treatment of depression faces several technical limitations. Firstly, the lack of standardized treatment and evaluation protocols hinders the ability to draw clear conclusions about its efficacy. Secondly, the mechanisms underlying the interactions between pathogens and beneficial bacteria in the context of depression are not fully understood. Thirdly, the safety and potential ethical issues associated with FMT, especially during the transition from clinical trials to clinical application, need to be carefully considered. These challenges highlight the importance of strengthening technical and methodological research to optimize FMT protocols and ensure its safe and effective use in depression treatment.

### 4.1 Limitations and advantages

There are some obvious limitations in our current study, which are mainly reflected in the following key aspects: First, it is challenging to determine whether FMT significantly differs between healthy individuals and patients with depression because of the small sample size of the particular population group with a diagnosis of depression. Our thorough comprehension of FMT requirements and the impact of interventions in both population groups was hampered by this restriction. Second, after subgroup analysis, the systematic examination revealed considerable inter-study heterogeneity, and we need to take into account additional possible sources of heterogeneity. Numerous factors, such as the genetic makeup of intestinal microbes, stress exposure, lifestyle, eating habits, gastrointestinal disorders, and drug use, may significantly affect the experimental outcomes for both FMT donors and recipients. It is important to note that the studies that were part of the systematic evaluation were carried out in different populations. It is more difficult for us to correctly evaluate the experimental results because of the variety and complexity of these variable components. Furthermore, it is impossible to overlook the difficulties brought about by the variety of study designs. The study adhered to the FMT protocol (intervention dose, donor source, repeated administration, route, etc.), but the depression assessment scale might introduce bias into the explanation of the FMT on the connection between depression and health, which could result in results that are not comparable. Lastly, it is impossible to quantify the relationship between drug usage and gut microbes, particularly the use of probiotics and antibiotics, if the study group includes people with other gastrointestinal disorders or other underlying conditions. Depression, on the other hand, is a type of psychological cognitive behavior that is influenced by a variety of elements, including quality of life, stress in life, and social relationships. This systematic review is comparatively biased and only looks at how FMT affects depression symptoms.

By combining information from existing research, we provide a thorough and current evaluation of randomized controlled trials that use FMT. In order to resolve the discrepancies in earlier research, our findings not only corroborate the impacts seen in earlier meta-analyses but also support similar conclusions in large-scale contemporary datasets. The comprehensive methodological quality evaluation and broad research scope of our review and meta-analysis illustrate its strengths.

## 5 Conclusion

Overall, the available data do not conclusively show that FMT is directly linked to a notable reduction in depressed symptoms. Nonetheless, our subgroup analyses suggest that antidepressant treatment with higher doses of FMT is still significant and desirable in patients with established depression. Given the paucity of relevant RCTs in patients with established depression, FMT needs to be further validated to be recommended as a reliable antidepressant treatment in the clinic. Although the extent and clinical significance of this impact have not been conclusively established, FMT may exhibit a particular demographic tendency toward perhaps alleviating depression symptoms.

To more accurately assess the efficacy of FMT in treating depressive symptoms, we still need to conduct more in-depth and standardized research. In future randomized controlled trials, we should employ more clearly defined patient populations and standardized outcome measures to establish robust evidence for the therapeutic effects of FMT and provide scientific justification for its potential application in treating depression.

## References

[1] O'Sullivan D Gordon BR Lyons M Meyer JD Herring MP . Effects of resistance exercise training on depressive symptoms among young adults: A randomized controlled trial. Psychiatry Res. (2023) 326:115322. doi: 10.1016/j.psychres.2023.115322, PMID: 37429171 PMC12309288

[2] Ferrari AJ Charlson FJ Norman RE Patten SB Freedman G Murray CJ . Burden of depressive disorders by country, sex, age, and year: findings from the global burden of disease study 2010. PloS Med. (2013) 10:e1001547. doi: 10.1371/journal.pmed.1001547, PMID: 24223526 PMC3818162

[3] Mathers CD Loncar D . Projections of global mortality and burden of disease from 2002 to 2030. PloS Med. (2006) 3:e442. doi: 10.1371/journal.pmed.0030442, PMID: 17132052 PMC1664601

[4] Noetel M Sanders T Gallardo-Gómez D Taylor P Del Pozo Cruz B van den Hoek D . Effect of exercise for depression: systematic review and network meta-analysis of randomized controlled trials. Bmj. (2024) 384:e075847. doi: 10.1136/bmj-2023-075847, PMID: 38355154 PMC10870815

[5] Østergaard SD Hieronymus F . Psilocybin for treatment-resistant depression. N Engl J Med. (2023) 388:e22. doi: 10.1056/NEJMc2215459, PMID: 36812443

[6] Vasiliadis HM Dionne PA Préville M Gentil L Berbiche D Latimer E . The excess healthcare costs associated with depression and anxiety in elderly living in the community. Am J Geriatr Psychiatry. (2013) 21:536–48. doi: 10.1016/j.jagp.2012.12.016, PMID: 23567409

[7] Chinna Meyyappan A Forth E Wallace CJK Milev R . Effect of fecal microbiota transplant on symptoms of psychiatric disorders: a systematic review. BMC Psychiatry. (2020) 20:299. doi: 10.1186/s12888-020-02654-5, PMID: 32539741 PMC7294648

[8] Cheng L Wu H Cai X Zhang Y Yu S Hou Y . A Gpr35-tuned gut microbe-brain metabolic axis regulates depressive-like behavior. Cell Host Microbe. (2024) 32:227–43.e6. doi: 10.1016/j.chom.2023.12.009, PMID: 38198925

[9] Cussotto S Clarke G Dinan TG Cryan JF . Psychotropics and the microbiome: a chamber of secrets. Psychopharmacol (Berl). (2019) 236:1411–32. doi: 10.1007/s00213-019-5185-8, PMID: 30806744 PMC6598948

[10] Foster JA McVey Neufeld KA . Gut-brain axis: how the microbiome influences anxiety and depression. Trends Neurosci. (2013) 36:305–12. doi: 10.1016/j.tins.2013.01.005, PMID: 23384445

[11] Zhang Q Bi Y Zhang B Jiang Q Mou CK Lei L . Current landscape of fecal microbiota transplantation in treating depression. Front Immunol. (2024) 15:1416961. doi: 10.3389/fimmu.2024.1416961, PMID: 38983862 PMC11231080

[12] Borkent J Ioannou M Laman JD Haarman BCM Sommer IEC . Role of the gut microbiome in three major psychiatric disorders. Psychol Med. (2022) 52:1222–42. doi: 10.1017/s0033291722000897, PMID: 35506416 PMC9157303

[13] Zhao W Lei J Ke S Chen Y Xiao J Tang Z . Fecal microbiota transplantation plus tislelizumab and fruquintinib in refractory microsatellite stable metastatic colorectal cancer: an open-label, single-arm, phase II trial (RENMIN-215). EClinicalMedicine. (2023) 66:102315. doi: 10.1016/j.eclinm.2023.102315, PMID: 38024475 PMC10679864

[14] Joo MK Lee JW Woo JH Kim HJ Kim DH Choi JH . Regulation of colonic neuropeptide Y expression by the gut microbiome in patients with ulcerative colitis and its association with anxiety- and depression-like behavior in mice. Gut Microbes. (2024) 16:2319844. doi: 10.1080/19490976.2024.2319844, PMID: 38404132 PMC10900276

[15] Doll JPK Vázquez-Castellanos JF Schaub AC Schweinfurth N Kettelhack C Schneider E . Fecal microbiota transplantation (FMT) as an adjunctive therapy for depression-case report. Front Psychiatry. (2022) 13:815422. doi: 10.3389/fpsyt.2022.815422, PMID: 35250668 PMC8891755

[16] Beurel E Toups M Nemeroff CB . The bidirectional relationship of depression and inflammation: double trouble. Neuron. (2020) 107:234–56. doi: 10.1016/j.neuron.2020.06.002, PMID: 32553197 PMC7381373

[17] Howes OD Thase ME Pillinger T . Treatment resistance in psychiatry: state of the art and new directions. Mol Psychiatry. (2022) 27:58–72. doi: 10.1038/s41380-021-01200-3, PMID: 34257409 PMC8960394

[18] Dailey FE Turse EP Daglilar E Tahan V . The dirty aspects of fecal microbiota transplantation: a review of its adverse effects and complications. Curr Opin Pharmacol. (2019) 49:29–33. doi: 10.1016/j.coph.2019.04.008, PMID: 31103793

[19] Page MJ McKenzie JE Bossuyt PM Boutron I Hoffmann TC Mulrow CD . The PRISMA 2020 statement: an updated guideline for reporting systematic reviews. Bmj. (2021) 372:n71. doi: 10.1136/bmj.n71, PMID: 33782057 PMC8005924

[20] Higgins JP Altman DG Gøtzsche PC Jüni P Moher D Oxman AD . The Cochrane Collaboration's tool for assessing risk of bias in randomized trials. Bmj. (2011) 343:d5928. doi: 10.1136/bmj.d5928, PMID: 22008217 PMC3196245

[21] Cheng Y Tan G Zhu Q Wang C Ruan G Ying S . Efficacy of fecal microbiota transplantation in patients with Parkinson's disease: clinical trial results from a randomized, placebo-controlled design. Gut Microbes. (2023) 15:2284247. doi: 10.1080/19490976.2023.2284247, PMID: 38057970 PMC10841011

[22] Lin H Guo Q Wen Z Tan S Chen J Lin L . The multiple effects of fecal microbiota transplantation on diarrhea-predominant irritable bowel syndrome (IBS-D) patients with anxiety and depression behaviors. Microb Cell Fact. (2021) 20:233. doi: 10.1186/s12934-021-01720-1, PMID: 34963452 PMC8715582

[23] Lahtinen P Jalanka J Hartikainen A Mattila E Hillilä M Punkkinen J . Randomized clinical trial: fecal microbiota transplantation versus autologous placebo administered via colonoscopy in irritable bowel syndrome. Aliment Pharmacol Ther. (2020) 51:1321–31. doi: 10.1111/apt.15740, PMID: 32343000

[24] Mazzawi T Hausken T Refsnes PF Hatlebakk JG Lied GA . The effect of anaerobically cultivated human intestinal microbiota compared to fecal microbiota transplantation on gut microbiota profile and symptoms of irritable bowel syndrome, a double-blind placebo-controlled study. Microorganisms. (2022) 10:1819. doi: 10.3390/microorganisms10091819, PMID: 36144420 PMC9503104

[25] Ghorbani Y Schwenger KJP Sharma D Jung H Yadav J Xu W . Effect of fecal microbial transplant via colonoscopy in patients with severe obesity and insulin resistance: A randomized double-blind, placebo-controlled Phase 2 trial. Diabetes Obes Metab. (2023) 25:479–90. doi: 10.1111/dom.14891, PMID: 36239189

[26] Green JE Berk M Mohebbi M Loughman A McGuinness AJ Castle D . Feasibility, acceptability, and safety of fecal microbiota transplantation in the treatment of major depressive disorder: A pilot randomized controlled trial. Can J Psychiatry. (2023) 68:315–26. doi: 10.1177/07067437221150508, PMID: 36637229 PMC10192831

[27] Aroniadis OC Brandt LJ Oneto C Feuerstadt P Sherman A Wolkoff AW . Fecal microbiota transplantation for diarrhea-predominant irritable bowel syndrome: a double-blind, randomized, placebo-controlled trial. Lancet Gastroenterol Hepatol. (2019) 4:675–85. doi: 10.1016/s2468-1253(19)30198-0, PMID: 31326345

[28] Simpson CA Diaz-Arteche C Eliby D Schwartz OS Simmons JG Cowan CSM . The gut microbiota in anxiety and depression -A systematic review. Clin Psychol Rev. (2021) 83:101943. doi: 10.1016/j.cpr.2020.101943, PMID: 33271426

[29] Šuler Baglama Š Trčko K . Skin and gut microbiota dysbiosis in autoimmune and inflammatory skin diseases. Acta Dermatovenerol Alp Pannonica Adriat. (2022) 31:105–9. doi: 10.15570/actaapa.2022.16, PMID: 36149040

[30] Sudo N Chida Y Aiba Y Sonoda J Oyama N Yu XN . Postnatal microbial colonization programs the hypothalamic-pituitary-adrenal system for stress response in mice. J Physiol. (2004) 558:263–75. doi: 10.1113/jphysiol.2004.063388, PMID: 15133062 PMC1664925

[31] Diaz Heijtz R Wang S Anuar F Qian Y Björkholm B Samuelsson A . Normal gut microbiota modulates brain development and behavior. Proc Natl Acad Sci U S A. (2011) 108:3047–52. doi: 10.1073/pnas.1010529108, PMID: 21282636 PMC3041077

[32] Neufeld KM Kang N Bienenstock J Foster JA . Reduced anxiety-like behavior and central neurochemical change in germ-free mice. Neurogastroenterol Motil. (2011) 23:255–64, e119. doi: 10.1111/j.1365-2982.2010.01620.x, PMID: 21054680

[33] Perez-Pardo P Dodiya HB Engen PA Forsyth CB Huschens AM Shaikh M . Role of TLR4 in the gut-brain axis in Parkinson's disease: a translational study from men to mice. Gut. (2019) 68:829–43. doi: 10.1136/gutjnl-2018-316844, PMID: 30554160

[34] Liang S Wu X Hu X Wang T Jin F . Recognizing depression from the microbiota-Gut-Brain axis. Int J Mol Sci. (2018) 19:1592. doi: 10.3390/ijms19061592, PMID: 29843470 PMC6032096

[35] Kelly JR Kennedy PJ Cryan JF Dinan TG Clarke G Hyland NP . Breaking down the barriers: the gut microbiome, intestinal permeability and stress-related psychiatric disorders. Front Cell Neurosci. (2015) 9:392. doi: 10.3389/fncel.2015.00392, PMID: 26528128 PMC4604320

[36] Alexandrov PN Hill JM Zhao Y Bond T Taylor CM Percy ME . Aluminum-induced generation of lipopolysaccharide (LPS) from the human gastrointestinal (GI)-tract microbiome-resident Bacteroides fragilis. J Inorg Biochem. (2020) 203:110886. doi: 10.1016/j.jinorgbio.2019.110886, PMID: 31707334 PMC7409391

[37] Miller AH Haroon E Felger JC . Therapeutic implications of brain-immune interactions: treatment in translation. Neuropsychopharmacology. (2017) 42:334–59. doi: 10.1038/npp.2016.167, PMID: 27555382 PMC5143492

[38] Danne C Skerniskyte J Marteyn B Sokol H . Neutrophils: from IBD to the gut microbiota. Nat Rev Gastroenterol Hepatol. (2024) 21:184–97. doi: 10.1038/s41575-023-00871-3, PMID: 38110547

[39] Lee SM Dong TS Krause-Sorio B Siddarth P Milillo MM Lagishetty V . The intestinal microbiota as a predictor for antidepressant treatment outcome in geriatric depression: a prospective pilot study. Int Psychogeriatr. (2022) 34:33–45. doi: 10.1017/s1041610221000120, PMID: 33757609

[40] Raes J . Nifty new tools for microbiome treatment design. Nat Rev Gastroenterol Hepatol. (2023) 20:77–8. doi: 10.1038/s41575-022-00735-2, PMID: 36609547

[41] Yang R Chen Z Cai J . Fecal microbiota transplantation: Emerging applications in autoimmune diseases. J Autoimmun. (2023) 141:103038. doi: 10.1016/j.jaut.2023.103038, PMID: 37117118

[42] Qu Z Tian P Yang B Zhao J Wang G Chen W . Fecal microbiota transplantation for diseases: Therapeutic potential, methodology, risk management in clinical practice. Life Sci. (2022) 304:120719. doi: 10.1016/j.lfs.2022.120719, PMID: 35716734

[43] Liu X Liu M Zhao M Li P Gao C Fan X . Fecal microbiota transplantation for the management of autoimmune diseases: Potential mechanisms and challenges. J Autoimmun. (2023) 141:103109. doi: 10.1016/j.jaut.2023.103109, PMID: 37690971

[44] Cammarota G Ianiro G Tilg H Rajilić-Stojanović M Kump P Satokari R . European consensus conference on fecal microbiota transplantation in clinical practice. Gut. (2017) 66:569–80. doi: 10.1136/gutjnl-2016-313017, PMID: 28087657 PMC5529972

[45] Bokoliya SC Dorsett Y Panier H Zhou Y . Procedures for fecal microbiota transplantation in murine microbiome studies. Front Cell Infect Microbiol. (2021) 11:711055. doi: 10.3389/fcimb.2021.711055, PMID: 34621688 PMC8490673

