# Supplementary material for: Efficacy effects of fecal microbiota transplantation on depressive symptoms: a meta-analysis based on randomized controlled trials
Source: Front Psychiatry. 2026 Jan 29;16:1629290. doi: 10.3389/fpsyt.2025.1629290 (PMC12895053; doi:10.3389/fpsyt.2025.1629290)
Supplement: Supplementary file 1 [file Table1.docx]

| Supplementary material  **Table S1**. Search strategies for the databases. | | |
| --- | --- | --- |
| Database  (Search date) | Search strategy | Number of results |
| PubMed | TA=depression OR depressive disorder OR depressive disorders OR major depression OR depressive disorder OR depressed individuals OR major depressive disorder OR MDD OR dysthymic disorder OR suicidal OR suicide OR anxiety OR well-being OR wellbeing OR negative emotion OR quality of life OR self esteem OR self-esteem OR mental health OR mental disorders OR mental disorder OR psychological distress OR self efficacy OR self-efficacy OR resilience OR empowerment OR anxiety depression OR depression anxiety OR mood disorders OR life skills OR anhedonia OR resilience OR emotional OR sadness OR psychology OR loss of interest OR participation OR psychological distress OR mental capital OR restless OR psychosocial OR psychiatry OR suicid OR suicide OR melancholia OR [seasonal affective disorder](https://www.ncbi.nlm.nih.gov/mesh/68016574) OR [dysthymic disorder](https://www.ncbi.nlm.nih.gov/mesh/68019263) | 1,923,514 |
|  | TA=Bacteriotherapy OR Microbial Therapy OR Microbe Therapy OR Fecal Microbiota Transplantation OR Feces infus OR Fecal infus OR Microbiota infus OR Stool infus OR Microbiome infus OR Microflor infus OR Feces transplant OR Fecal transplant OR Microbiota transplant OR mental disorders OR mental disorder OR psychological distress OR Stool transplant OR Microbiome transplant OR Microflor transplant OR Feces enema OR Fecal enema OR Microbiota enema OR Stool enema OR Microbiome enema OR Microflor enema OR Feces donor OR Fecal donor OR Microbiota donor OR Stool donor OR Microbiome donor OR Microflor donor OR FMT | 104,021 |
|  | RCT OR randomized controlled trial OR randomized controlled OR placebo OR placebos | 519,280 |
|  | 1# AND #2 AND #3 | 1671 |
| WOS | TS=depression OR depressive disorder OR depressive disorders OR major depression OR depressive disorder OR depressed individuals OR major depressive disorder OR MDD OR dysthymic disorder OR suicidal OR suicide OR anxiety OR well-being OR wellbeing OR negative emotion OR quality of life OR self esteem OR self-esteem OR mental health OR mental disorders OR mental disorder OR psychological distress OR self efficacy OR self-efficacy OR resilience OR empowerment OR anxiety depression OR depression anxiety OR mood disorders OR life skills OR anhedonia OR resilience OR emotional OR sadness OR psychology OR loss of interest OR participation OR psychological distress OR mental capital OR restless OR psychosocial OR psychiatry OR suicid OR suicide OR melancholia OR [seasonal affective disorder](https://www.ncbi.nlm.nih.gov/mesh/68016574) OR [dysthymic disorder](https://www.ncbi.nlm.nih.gov/mesh/68019263) | 1,611,199 |
|  | TS=Bacteriotherapy OR Microbial Therapy OR Microbe Therapy OR Fecal Microbiota Transplantation OR Feces infus OR Fecal infus OR Microbiota infus OR Stool infus OR Microbiome infus OR Microflor infus OR Feces transplant OR Fecal transplant OR Microbiota transplant OR mental disorders OR mental disorder OR psychological distress OR Stool transplant OR Microbiome transplant OR Microflor transplant OR Feces enema OR Fecal enema OR Microbiota enema OR Stool enema OR Microbiome enemaOR Microflor enema OR Feces donor OR Fecal donor OR Microbiota donor OR Stool donor OR Microbiome donor OR Microflor donor OR FMT | 169,825 |
|  | TS=RCT OR randomized controlled trial OR randomized controlled OR placebo OR placebos | 528,178 |
|  | 1# AND #2 AND #3 | 908 |
| Medline | depression OR depressive disorder OR depressive disorders OR major depression OR depressive disorder OR depressed individuals OR major depressive disorder OR MDD OR dysthymic disorder OR suicidal OR suicide OR anxiety OR well-being OR wellbeing OR negative emotion OR quality of life OR self esteem OR self-esteem OR mental health OR mental disorders OR mental disorder OR psychological distress OR self efficacy OR self-efficacy OR resilience OR empowerment OR anxiety depression OR depression anxiety OR mood disorders OR life skills OR anhedonia OR resilience OR emotional OR sadness OR psychology OR loss of interest OR participation OR psychological distress OR mental capital OR restless OR psychosocial OR psychiatry OR suicid OR suicide OR melancholia OR seasonal affective disorder OR dysthymic disorder | 3,676,820 |
|  | Bacteriotherapy OR Microbial Therapy OR Microbe Therapy OR Fecal Microbiota Transplantation OR Feces infus OR Fecal infus OR Microbiota infus OR Stool infus OR Microbiome infus OR Microflor infus OR Feces transplant OR Fecal transplant OR Microbiota transplant OR mental disorders OR mental disorder OR psychological distress OR Stool transplant OR Microbiome transplant OR Microflor transplant OR Feces enema OR Fecal enema OR Microbiota enema OR Stool enema OR Microbiome enemaOR Microflor enema OR Feces donor OR Fecal donor OR Microbiota donor OR Stool donor OR Microbiome donor OR Microflor donor OR FMT | 103,728 |
|  | RCT OR randomized controlled trial OR randomized controlled OR placebo OR placebos | 650,860 |
|  | 1# AND #2 AND #3 | 562 |
| Cochrane library | depression OR depressive disorder OR depressive disorders OR major depression OR depressive disorder OR depressed individuals OR major depressive disorder OR MDD OR dysthymic disorder OR suicidal OR suicide OR anxiety OR well-being OR wellbeing OR negative emotion OR quality of life OR self esteem OR self-esteem OR mental health OR mental disorders OR mental disorder OR psychological distress OR self efficacy OR self-efficacy OR resilience OR empowerment OR anxiety depression OR depression anxiety OR mood disorders OR life skills OR anhedonia OR resilience OR emotional OR sadness OR psychology OR loss of interest OR participation OR psychological distress OR mental capital OR restless OR psychosocial OR psychiatry OR suicid OR suicide OR melancholia OR [seasonal affective disorder](https://www.ncbi.nlm.nih.gov/mesh/68016574) OR [dysthymic disorder](https://www.ncbi.nlm.nih.gov/mesh/68019263) | 1,229,967 |
|  | Bacteriotherapy OR Microbial Therapy OR Microbe Therapy OR Fecal Microbiota Transplantation OR Feces infus OR Fecal infus OR Microbiota infus OR Stool infus OR Microbiome infus OR Microflor infus OR Feces transplant OR Fecal transplant OR Microbiota transplant OR mental disorders OR mental disorder OR psychological distress OR Stool transplant OR Microbiome transplant OR Microflor transplant OR Feces enema OR Fecal enema OR Microbiota enema OR Stool enema OR Microbiome enemaOR Microflor enema OR Feces donor OR Fecal donor OR Microbiota donor OR Stool donor OR Microbiome donor OR Microflor donor OR FMT | 3,260 |
|  | RCT OR randomized controlled trial OR randomized controlled OR placebo OR placebos | 1,229,863 |
|  | 1# AND #2 AND #3 | 1457 |

**
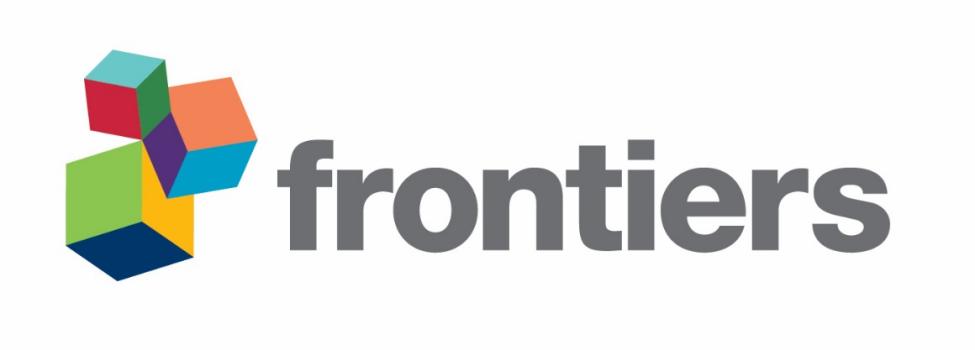
**
